# Prevalence and demographic profile of keratoconus among high school students in Kenya

**DOI:** 10.1007/s10792-024-03370-9

**Published:** 2025-01-09

**Authors:** Zahra Aly Rashid, Khathutshelo Percy Mashige, Vanessa Raquel Moodley

**Affiliations:** Discipline of Optometry, School of Health Sciences, University of KwaZulu-Natal, Durban, South Africa

**Keywords:** Keratoconus, Prevalence, Kenya, Africa, High school, Adolescents

## Abstract

**Purpose:**

To determine the prevalence and demographic profile of keratoconus (KC) among high school students in Nairobi County, Kenya.

**Methods:**

In this population-based, prospective, cross-sectional study, multistage cluster sampling was used to select the participants. All students underwent visual acuity measurement, auto-refraction, retinoscopy and corneal topography. Students with a scissors reflex on retinoscopy or corneal topography patterns suspicious of KC were referred for corneal tomography.

**Results:**

A total of 3051 students from 29 schools, with a mean age of 17.4 ± 1.6 years (range: 13–25 years) were screened. The prevalence of KC was 1.7% (*n* = 51) (95% CI, 1.2–2.2) and of KC suspects was 3.8% (*n* = 117) (95% CI, 3.2–4.6). There were no significant associations between the prevalence of KC and gender, age or ethnicity (all *p* > 0.05). In the KC group, 88.2% (*n* = 45) were unaware of their condition, 52.9% (*n* = 27) were treated for allergic conjunctivitis, 45.1% (*n* = 23) required spectacles, 11.8% (*n* = 6) were recommended contact lenses in at least one eye and 49.0% (*n* = 25) were recommended corneal cross-linking.

**Conclusions:**

Among adolescents, the results of this study indicate a higher prevalence of KC compared to that reported in South Korea, Norway, Brazil and Caucasians in New Zealand, but less than that reported in some countries in the Middle East. Given the high prevalence revealed in this study, a national school screening program and clinical guidelines for screening, diagnosis and management of KC is recommended.

## Introduction

Keratoconus (KC) is a corneal disorder that usually presents in adolescence. The prevalence of KC in Western countries [1–5] and North and East Asia [6–9] is generally < 1.3% with one exception where Chan et al. reported a prevalence of 3.4% in Australia in a predominantly Caucasian cohort [10]. In Turkey, India and the Middle East the prevalence ranges from 1.4 to 4.8% [11–14]. Previously KC was considered quite rare, however, increased rates of the disease are being reported in recent studies. A meta-analysis of a limited number of studies by Akowuah et al. [15], estimated the prevalence of KC to be 7.9% in Africa. The wide variation in prevalence rates can be explained by sample size differences, inconsistency in the gender ratio of subjects, use of different diagnostic tools and methods, a lack of an accurate definition for KC, selection bias and ethnic and environmental factors [16].

KC is reported to generally develop between 20 and 30 years of age but can develop in pre-teens [17], adolescence [18] or later in life [6]. Some studies have shown a higher prevalence of KC in males and others in females, whilst some have shown no difference [16].

Detection and diagnosis of KC occurs with the use of a combination of tools namely retinoscopy, keratometry, slit lamp biomicroscopy, corneal topography, pachymetry and corneal tomography [19]. Non-surgical management is with spectacles in mild to moderate cases and specialized contact lenses (CL) in moderate to advanced KC. Surgical options include corneal cross-linking (CXL) to halt the progression in mild to moderate stages and corneal transplants [20] in advanced to severe stages.

As a progressive disease, a delay in diagnosing KC has a negative impact on the vision-related quality of life (QOL); making early diagnosis a key to halting the progression and preventing visual loss [21].

Unpublished reports from clinicians suggest that the prevalence of KC in adolescents in Kenya is high and that many present with advanced stages of the disease. The country has no national school screening program and hence many children are not diagnosed with KC and referred early enough for treatment. Those who are self-referred may often still not be correctly diagnosed due to the lack of specialized paediatric refractive and diagnostic services [22]. If referred when KC has significantly progressed, the corneas may be too thin for CXL [23] and speciality CLs for vision rehabilitation may be required, which are not always available or affordable. If satisfactory visual acuity (VA) is not achieved with scleral CLs, a corneal transplant may be necessary. Corneal donor tissue is not easily available and can be costly, with many patients not returning for follow-up due to financial barriers [24].

The prevalence of KC in Kenya is currently unknown, as in most African countries, due to a shortage of population-based studies [15]. There is a need for epidemiological studies to understand the magnitude of KC across Africa. With limited research on KC in African eyes, management is guided by studies undertaken outside the continent, which may not be appropriate for patients of African descent. The study aimed to estimate the prevalence of KC in a high school population to better understand the burden of the disease and guide policymakers for better planning.

## Methods

This population-based, prospective, quantitative, cross-sectional cohort study was undertaken from the 25th October 2021 to the 26th August 2022. Using a stratified cluster random sampling strategy, 7567 out of 24,348 high school students in four of eleven sub-counties in Nairobi were invited to participate.

Demographic information on age, gender, grade and self-reported ethnicity was collected. All students underwent VA assessment, objective refraction and corneal topography at school. The screening team consisted of four optometrists and three non-clinical staff who received training in the use of the equipment and the research protocol. VA was measured at 3 m with a Lea Numbers 13-line translucent distance chart (#271,200, Good-Lite Co., Chicago, IL, USA), in a brightly lit part of the classroom and recorded in Logarithm of the Minimum Angle of Resolution (logMAR) notation. Unaided and aided monocular VAs were measured. If three out of five numbers on the 0.2 logMAR line were read, the VA was noted as 0.2 logMAR and further smaller lines were not attempted. The 2WIN-S auto-refractor (Adaptica, Padova, Italy) and a Keeler Professional streak retinoscope (Keeler, Windsor, UK) were used for objective refraction. The presence of a scissors reflex was noted. Those who had a significant refractive error on retinoscopy of ≤ −0.75DS, ≥  + 1.50DS and/or ≤ −0.75DC were referred for subjective refraction and dispensed spectacles. Students with irregular patterns except for symmetric bowtie, oval or round patterns with the hand-held EyeSys Vista (Version 2.6, EyeSys, Houston, TX, USA) corneal topographer were also tested with the Medmont E-300 (Medmont International, Victoria, Australia), conducted in school. All those who had a scissors reflex on retinoscopy or an Inferior-Superior (I-S) index ≥ 1.0 or had an I-S index < 1.0 but scored 5 out of 6 points on the indices shown in Table 1, were referred for corneal tomography with the MS-39 AS-OCT (CSO, Scandicci, Italy) at City Eye Hospital (CEH). Students were classified as non-keratoconus (NKC), keratoconus suspect (KCS) and KC based on clinical signs and corneal tomography.

**Table 1:** Indices used on the Medmont topographer as referral criteria

Parameter	Cut-off	Points
SAI	≥ 0.7	1
SRI	≥ 0.6	1
Flat e	> 0.7	1
Corneal power difference (D)	> 10	1
Corneal topography pattern that was not round/oval/symmetrical bowtie	–	1
Scissors reflex on retinoscopy	Yes	1

*SAI* surface asymmetry index, *SRI* surface regularity index, *e* eccentricity

### Statistical analysis

Percentages and 95% confidence intervals were reported for categorical data and Chi-square tests or Fisher’s exact tests were used to compare the three groups; namely NKC, KCS and KC. Stata V17 statistical software was used in the analysis and *p* < 0.05 was considered as statistically significant.

## Results

**Fig. 1:**
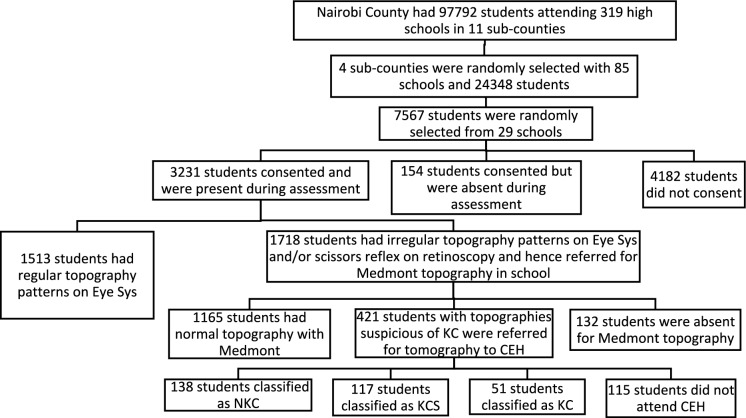
The process from selection to screening of students

Of the 7567 students invited to participate, 3385 responded (response rate = 44.7%), out of which 154 were absent during screening. A total of 3231 students from 29 schools were screened. The records of 180 students were excluded because they were not Kenyan residents, had ocular pathology other than KC or were referred to CEH but did not attend (Fig. 1).

The data of 3051 students who were assessed at school were analysed. Their mean age was 17.4 ± 1.6 years (range: 13–25 years) and there were 57.3% (*n* = 1749) females and 77.5% (*n* = 2 363) from public schools.

Table 2 presents the prevalence of KC according to gender, type of school, age group, and ethnicity. The prevalence of KC was 1.7% (*n* = 51) (95% CI, 1.2–2.2) and of KCS was 3.8% (*n* = 117) (95% CI, 3.2–4.6). The prevalence of KC was not significantly associated with gender (*p* = 0.80), type of school (*p* = 0.90), age (*p* = 0.30) or ethnicity (*p* = 0.50).

**Table 2:** The prevalence of keratoconus according to the demographic profile of participants

	Total (n)	Keratoconic (n)	Prevalence (%, 95% CI)	*p* value
	3051	51	1.7[1.2, 2.2]	
*Gender*				
Male	1302	21	1.6[1.0, 2.5]	0.80
Female	1749	30	1.7[1.2, 2.4]	
*Type of school*				
Public	2363	40	1.7[1.2, 2.3]	0.90
Private	688	11	1.6[0.8, 2.8]	
*Age (years)*				
13–14	86	3	3.5[0.7, 9.9]	0.30
15–16	850	16	1.9[1.1, 3.0]	
17–18	1386	18	1.3[0.8, 2.0]	
≥ 19	729	14	1.9[1.1, 3.2]	
*Ethnicity*				
Kikuyu	1038	16	1.5[0.9, 2.5]	0.50
Luo	600	8	1.3[0.6, 2.6]	
Luhya	442	9	2.0[0.9, 3.8]	
Kamba	355	3	0.8[0.2, 2.4]	
Kenyan Somali	250	5	2.0[0.7, 4.6]	
Kisii	136	3	2.2[0.5, 6.3]	
Others (n < 50)	230	7	3.0[1.2, 6.2]	

Overall, 10.7% (*n* = 326) of students needed spectacles and 3.7% (*n* = 114) needed eye drops for the management of allergic conjunctivitis. In the KC group, 88.2% (*n* = 45) were unaware of their condition, 52.9% (*n* = 27) were treated for allergic conjunctivitis, 45.1% (*n* = 23) had significant refractive errors and required spectacles, 11.8% (*n* = 6) were recommended CLs in at least one eye and 49.0% (*n* = 25) were recommended CXL.

## Discussion

This is the first population-based study in Kenya to estimate the prevalence of KC and confirms the anecdotal observations of a high prevalence of the disease among high school students. Our study found the prevalence of KC and KCS to be 1.7% and 3.8%, respectively.

As expected, we found a lower prevalence of KC compared to facility-based studies previously reported in Africa [15] which included high-risk individuals with either allergic conjunctivitis [25, 26] or astigmatism [27] or seeking refractive surgery [28, 29] or CL services [30]. One exception was a study in Ghana, where Kobia-Acquah et al. reported a prevalence of < 1%, possibly due to low uptake of referrals, under-diagnosis and/or misdiagnosis of the condition [31].

When compared with other studies conducted among adolescents, the prevalence of KC in Kenyans was lower than that found in Saudi Arabians (2.8–4.8%) [14, 32], similar to that in Mexicans (1.8%) [33], Maori islanders (2.3%) [34] and Emiratis (2.7%) [18] but higher than that in Brazilians (0.7%) [35], Caucasians (0.52%) in New Zealand and Norway (0.2%) [34, 36] and Asians in South Korea (0.06%) [6]. The variation in the prevalence of KC in these studies could be explained by differences in a combination of factors such as ethnicity, consanguinity, sunlight exposure, ocular allergy or corneal thickness.

Ethnicity, rather than geographical location, is likely to affect the prevalence of KC. Maori and Pacific ethnicities in New Zealand [34, 37] and Latinos and Blacks in the US [4, 38] were found to have a higher prevalence of KC than Caucasians living in the same country. This implies the contribution of genetic and heredity factors in the aetiology of the disease [39–41].

Consanguinity is a traditional practice in countries that form a belt spanning from Morocco to Pakistan across North Africa, the Middle East and Western Asia, including South India ranging from 20 to 50% [42] of all marriages which could account for the high prevalence of KC in these populations [41, 43, 44]. The prevalence of consanguinity across most of Africa is generally unknown [45], with one study conducted in Cameroon reporting a prevalence of 1.9% for first-cousin marriages [46].

The prevalence of KC appears to be higher in countries with hot and dry climates compared to cooler climates [47] which could explain the higher prevalence of KC in Kenya. It is thought that excessive exposure to UV light might cause oxidative stress to the cornea [48]. However, studies in animals [49, 50] and humans [51] have not shown a definitive association. Additionally, confounding variables such as ethnicity and genetics, make it difficult to assess its contribution towards the aetiology of KC.

Higher prevalence rates of vernal keratoconjunctivitis (VKC) have been reported in more African, Mediterranean and Middle Eastern countries than in Western countries [52]. In African children, the prevalence of VKC ranges from 4.0 to 39.9% [53–55]. In Kenya specifically, allergic conjunctivitis accounts for 27% of outpatient visits in eye clinics [56] and the prevalence of KC in children with allergic conjunctivitis was 30.9% [25]. In this study, more than half of the students with KC had allergic conjunctivitis and reported rubbing their eyes, similar to other studies in children [57]. Kerosene and firewood cooking, dust exposure and animal contact were reported to be significantly associated with VKC in Ethiopia [54, 55]. Ocular allergy is likely an indirect cause of KC and the eye rubbing caused by it is a risk factor for the development of KC and not the allergy itself [12, 58, 59]. Eye rubbing has both bio-mechanical and bio-cellular effects on the cornea. It is thought that the friction from eye rubbing increases epithelial temperature and intraocular pressure resulting in the collagen fibres and the proteoglycans slipping, deforming and thinning the cornea, causing it to lose rigidity and steepen [60, 61]. In addition, eye rubbing causes the release of proteolytic enzymes and inflammatory mediators from epithelial and stromal cells, resulting in the apoptosis of keratocytes, which releases enzymes that degrade the collagen fibrils and extracellular matrix leading to the onset/progression of KC [59, 62]. With such a high prevalence rate in the current study, it is advised that practitioners screen all children with VKC and that all those with a scissors reflex on retinoscopy be referred for corneal tomography. This will aid in early diagnosis and referral for CXL treatment to retard further development in those diagnosed with incipient KC. By managing the ocular allergy with medication and patient education, eye rubbing could be reduced and KC onset/progression prevented.

Similar to many studies in Africa [15], and around the world, we did not find a significant difference between the prevalence of KC and gender [1, 6, 11, 12]. However, there are conflicting results with some studies showing a higher prevalence in men [9, 10, 57, 63–65] and a few in women [2, 4, 13]. Sex hormones and hormonal imbalances in both males and females may play a role in the development of KC [66, 67].

The majority (n = 45; 88%) of students with KC were not aware of their diagnosis, despite half of them having moderate to severe KC on presentation, the reasons for which could be due to the complex nature of the disease, personal factors and the lack of affordable, accessible and quality paediatric eye care services [68]. Rono et al. reported that in Kenya there is a lack of awareness of eye conditions, low awareness of services available, low parental education and fear of costs associated with accessing services and interventions [69]. KC is asymptomatic in the early stages and asymmetric where one may not appreciate a reduction in vision until the better eye gets affected and there could be a short interval between noticeable symptoms and progression due to the ‘explosive’ nature of the disease in children [70]. Children find strategies to cope with reduced vision by sitting closer to the blackboard, squeezing their eyes or may not report mild visual impairment. Health-systems barriers include a lack of school screening programs, lack of insurance to cover spectacles and the lack of knowledge, skills and equipment among mid-level ophthalmic workers (MLOWs) to screen, diagnose and manage KC [22]. Including KC tests in school screening programmes and during routine eye exams, conducting awareness campaigns among students, teachers and parents, upskilling MLOWs and providing funding for spectacles and CXL in children would result in early identification and prompt management.

A strength of this study is that it is the first to report the prevalence of KC in Kenya and that the sample size was relatively large, randomly selected and representative of the national census ethnicity data. In addition, a final diagnosis of patients with KC was based on both corneal tomography and slit lamp examinations. There are some limitations to acknowledge. Firstly, the battery of tests used at school could not determine a posterior corneal profile and hence some children with posterior corneal steepening only may have been missed, affecting the final prevalence estimate. Secondly, more than half of the students did not return the consent forms. Strategies to increase participation need to be explored for future studies and more studies on KC prevalence in different parts of the country are recommended.

## Conclusions

This is the first study to describe the prevalence of KC in Kenya. Our findings demonstrate that approximately 1 in 50 high school students is likely to have the disease and screening for KC with a portable corneal topographer in schools is feasible. The higher prevalence of KC in Kenya could be attributed to a combination of genetics, high prevalence of ocular allergy and hence eye rubbing and thinner corneas. Given the high prevalence of this asymptomatic disease, with a significant proportion of students having VKC and the lack of specialised paediatric services, a national school screening program, KC screening, diagnosis and management guidelines for clinicians and patient and parent education is required. The implementation of these recommendations will result in early identification and timely intervention, preventing the need for long-term costly interventions and visual impairment that negatively affects the QOL of those with KC.

## Data Availability

No datasets were generated or analysed during the current study.
